# Global pattern in hunger and educational opportunity: a multilevel analysis of child hunger and TIMSS mathematics achievement

**DOI:** 10.1186/s40536-023-00161-z

**Published:** 2023-04-11

**Authors:** Yusuf Canbolat, David Rutkowski, Leslie Rutkowski

**Affiliations:** 1 Department of Education Policy Studies, Indiana University, 201 N. Rose Avenue, Bloomington, IN 47405 USA; 2 Department of Counseling and Educational Psychology, Indiana University, Bloomington, USA

## Abstract

In low-income countries, there exists a common concern about the effect of hunger and food insecurity on educational outcomes. However, income inequalities, economic slowdown, conflict, and climate change have raised those concerns globally. Yet, little is known about how widespread the problem of hunger in schools is worldwide. This study examines child hunger and student achievement internationally, using data from the Trends in Mathematics and Science Study (TIMSS) 2019. To examine the relationship between hunger and student achievement, we fitted multilevel models to the data and controlled for student SES, class SES, teacher experience, and teacher educational attainment.  The results suggest that hunger among students is not exclusive to low-income countries. Instead, child hunger is a common issue around the world, affecting about one-third of children and exacerbating unequal education opportunities globally. Controlling for other variables, the achievement gap between students who never come to school hungry and those who come to school always or almost always hungry is significant and deserves our attention. A clear policy recommendation from our results suggests that all countries that participated in TIMSS need to examine their school meal programs and explore ways to feed the students who show up to school hungry.

## Introduction

Food insecurity is the difficulty of accessing food due to limited resources. Often resulting in hunger, food insecurity is a global issue and affects about one-third of the world population (Cafiero et al., 2018; FAO, 2021b). Although food insecurity is most pervasive in low-income countries it is also prevalent in middle- and high-income countries. Acknowledging this global crisis, the United Nations placed the eradication of hunger and food insecurity by 2030 as its second Sustainable Development Goal (SDG2). Unfortunately, meeting this goal appears unlikely. The World Health Organization recently forecasted that the goal will be missed by a margin of nearly 660 million people, citing persistent income inequalities, economic slowdown exacerbated by the COVID-19 pandemic, conflict, and climate change as causes (FAO et al., 2021).

Difficulties in accessing adequate nutrition potentially hamper well-being for all; however, children tend to be the most vulnerable (UNICEF, 2020). For example, some of the wealthiest countries in the world such as the United Kingdom and the United States have one in five children that are food insecure. In other countries, the situation is even worse. In Chad, Kenya, Niger, Mozambique, Tanzania, and Uganda, more than three in five children experienced food insecurity (Pereira et al., 2017). Among other things, hunger may create persistent barriers to equal educational opportunity among children. Deficiencies in vitamins and minerals may reduce their mental concentration and cognition (Basch, 2011; Jensen, 2013). Poor nutrition may also weaken long-term brain development and memory (Frisvold, 2015). Further, students that are food insecure and go to school hungry often fail to fully participate in the learning process because they are distracted by hunger (Bogden et al., 2012).

In this paper, we explore the association between a major result of food insecurity, hunger, and academic achievement internationally. Specifically, using data from the Trends in Mathematics and Science Study (TIMSS) 2019, we examined the association between students who go to school hungry and their achievement in eighth-grade math. Within this analysis, we also study the achievement gap between food-insecure students and their peers and assess if that gap is confounded by socioeconomic status, peer effect, and teacher quality.

### Framework and literature review

Maslow's theory of the hierarchy of needs offers a relevant framework to understand the relationship between hunger and student achievement (Chinyoka, 2014). Maslow proposed that individuals seek to satisfy their needs based on a hierarchical model. More basic physical needs should be satisfied completely or substantially to reach higher-level needs such as cognitive, aesthetic, self-actualization, and transcendence needs (Gawel, 1996; Maslow, 1943). Therefore, if students are hungry, they will suppress all other higher-order needs, including active engagement in the learning process, to satisfy hunger since their motivational priority is hunger (Burleson & Thoron, 2014). Even if hungry students motivate themselves to engage in the learning process, they face fundamental physiological barriers preventing them from active participation in the learning process (Bogden et al., 2012; Frisvold, 2015). As a result, the frequency of hunger is expected to be associated with lower levels of learning and academic achievement.

Several studies found a link between hunger and lower academic achievement, especially among the young. For example, examining the relationship between hunger and learning across the life course, Aurino et al. (2020) found that hunger had a stronger negative effect on cognitive development in early childhood. Further, the repercussions of lacking proper meals in early childhood appears to be long-lasting. For example, research has shown that lacking food in early childhood resulted in disparities in educational attainment and achievement at later ages (Chakraborty & Jayaraman, 2019; Hinrichs, 2010). In other words, hunger can have both immediate and long-lasting effects on student ability. The relationship also seems to hold even after controlling for family background indicators (Lien, 2007; Metwally et al., 2020), suggesting that the negative relationship between hunger and achievement holds regardless of socioeconomic status and parental support. This relationship is prevalent across low-, middle-, and high-income countries. For example, in the Philippines, Glewwe et al. (2001) found that hunger and malnutrition reduced student achievement even after controlling for parental characteristics. In another study from Ethiopia, Seyoum et al. (2019) found that skipping breakfast was negatively associated with student achievement. And in Korea, Kim et al. (2003) found that the regularity of three meals (breakfast, lunch, and dinner) was associated with higher student achievement. Finally, in Norway, one of the world’s richest countries, Lien (2007) found that girls, children of less-educated, and immigrant families skipped breakfast more than boys and native students, which had an adverse influence on their educational achievement.

Oneway in which societies have attempted to assist students who come to school hungry is to fund schools so that they can provide meals for students. For example, in the US about 15 million students receive breakfast at school, and close to 30 million receive lunch every day (USDA, 2020). Of those students, the majority are provided the meals for free or at a discounted rate. The impetus for providing meals was largely informed by research that showed that those who faced challenges eating nutritious breakfast and lunch fell behind their peers in learning outcomes (Aurino et al., 2020; Glewwe et al., 2001; Kim et al., 2003; Seyoum et al., 2019). Further, studies that have evaluated reduced and free lunch programs found that these programs increased overall student achievement (Dotter, 2013; Frisvold, 2015). For example, in the US, Schwartz and Rothbart (2020) examined the effect of universal free lunch, extending free lunch to all students regardless of their income, on student achievement in New York City. The authors found that the program increased the achievement of non-poor students in math and language arts by about 0.08 standard deviations (SD) and 0.06 SD, respectively.

Other studies outside of the US also found a link between participation in free meals and greater academic achievement, both in the short and long term. For example, using a difference-in-differences approach, Fang and Zhu (2022) examined the effect of school nutrition programs on cognitive and health outcomes in China. The authors found that early exposure to the program increased test scores by 0.34 and 0.20 SD in reading and math respectively. Similarly, in Egypt, Metwally et al. (2020) used a matching approach to compare students who were exposed to a free meal program for five years and those who were not. After controlling for background, meal exposure was positively associated with higher achievement in math, but the relationship was modest for Arabic language achievement. Finally, in India, Chakraborty and Jayaraman (2019) examined the long-term effects of India’s free lunch program, the world’s largest free school meal program, on reading and math achievement among primary school students. The sample consisted of students in rural neighborhoods where nutritional deficiency was a common issue. Difference-in-differences results showed that relative to children who have less than a year of participation, exposure to the program for five years in elementary school increased achievement by 0.17 SD and 0.09 SD of reading and math achievement, respectively.

Examining the global prevalence of child food insecurity and hunger is challenging due to the lack of comprehensive surveys given to children. Therefore, existing research tends to rely on estimates based on household surveys that include children; however, the surveys are completed by adults within the household. For instance, the Food and Agriculture Organization of the United Nations (FAO) uses the Food Insecurity Experience Scale (FIES) to collect information about the frequency of food insecurity and hunger within a household because of lack of money or other resources. Using eight experience-based items such as “worried about enough food to eat”, “had to skip a meal”, and “ went without eating for a whole day”, FIES measures the severity of food insecurity among adults on the global scale (FAO, 2021a). Using the FIES measure in Gallup World Poll conducted in 147 countries, Pereira et al. (2017) found that 41% of children under the age of 15 live with a parent who has moderate or severe food insecurity. The researchers also reported that country income per capita was modestly correlated with food insecurity, suggesting that monetary poverty is not the sole driver. Studies highlighted that income inequalities, social welfare, and protection programs may shape food insecurity (Sandefur, 2022; WFP, 2020) implying that cross-country analysis of food insecurity should consider other relevant factors to better explain the issue.

Similar to the FIES, TIMSS collects experience-based information about hunger but differs in that it directly asks children, providing a unique opportunity to address the knowledge gaps around children that go to school hungry. In this paper, we aim to add to the literature by focusing on the following three interrelated research questions:

1. How does child hunger differ between countries and are differences associated with a country's wealth and income inequality?
2. What is the relationship between hunger and math achievement?
3. Does the relationship between hunger and math achievement change after controlling for student SES, class SES, teacher experience, and teacher educational attainment?

## Methods

### Data

TIMSS 2019 is a curriculum-based international assessment in mathematics and science. The target population is all students at the end of the fourth and eighth grades in participating educational systems. TIMSS also collects data from students, teachers, and principles of participating schools. For our analysis, we have limited our investigation to eighth-grade data because student hunger is relatively more prevalent at this grade, presenting more opportunity for analysis. On average in TIMSS 2019 countries, the proportions of students who report hunger every day or almost every day when they arrive at school are 33–28% in 8 and 4 grades, respectively.

According to Martin et al. (2020), TIMSS 2019 eighth-grade sample includes children ages 13 and 14 and is defined as the upper of the two adjacent grades with the most number of 13-year-olds. In 2019, 39 educational systems took place in the study. For this analysis, we used all 39 educational systems resulting in a sample of 227,345 students from 10,619 classes and 7483 schools. A two-stage stratified-cluster sample design was used. In the first stage, a sample of schools is selected among schools that have a target population of eighth-grade students. Explicit stratification by urbanization, region, and school size and implicit stratification (performance) is used at this stage. Then, in the second stage, one or more intact classes of students are selected from the sampled schools. Table 3 Appendix includes the list of educational systems analyzed for this study along with sample sizes and descriptive statistics for mathematics achievement and descriptive statistics for the variables used in our analysis which are described subsequently.

### Measures

To address our research questions, we used data from TIMSS 2019 eighth grade achievement scores and the student and teacher background questionnaire. We include teacher variables given empirical evidence, which we describe in the Analysis section. We also included gross domestic product per capita (The World Bank, 2022a) and the Gini coefficient (The World Bank, 2022b), which is a summary measure of income inequality, to explore the relationship between hunger and economic development across countries.

*Student Measures.* As a measure of hunger, we used the TIMSS 2019 student background question that asked students how often they felt hungry when they arrived at school. Students reported the frequency of hunger on a four-point Likert scale: never, sometimes, almost every day, and every day. In TIMSS 2019 international report, Mullis et al., (2020) aggregated the two hunger categories, every day and almost every day. We examined the relationship between the frequency of hunger and student achievement with and without aggregating these two response categories country-by-country (Table 4 Appendix). We found that the relationship is almost identical in all countries with and without aggregating those two response categories. These results suggested that aggregating those categories has no consequences for our relationship of interest. Thus, to be consistent with the TIMSS 2019 international report, the frequency of hunger consisted of three categories in our study: never (1), sometimes (2), and every day or almost every day (3) with higher values showing more frequent student hunger.

To control for student socioeconomic status (SES) in our models, we used a composite measure developed by the TIMSS study center that included the number of books in the student’s home, the highest level of education of either parent and the number of home study support (e.g., availability of internet connection and/or own room). According to Martin et al., (2020), students were scored based on their reports regarding the availability of the three resources. Cut scores were developed and divided into three the following three categories: “Students with Many Resources had a score at or above the cut score corresponding to reporting they had more than 100 books and both home study supports in their home and that at least one parent finished university, on average. Students with Few Resources had a score at or below the cut score corresponding to reporting they had 25 or fewer books and neither of the home study supports in the home and that neither parent had gone beyond upper secondary education, on average. All other students had Some Resources” (p. 289). Mullis et al. (2020) provided descriptive statistics and average achievement per category for all participating systems (p. 290–291).

As our outcome measure, we used overall student mathematics achievement, which is scaled, from the first cycle of the TIMSS assessment in 1995 to a mean of 500 and a standard deviation.

*Teacher Measures.* To help control differences in teacher quality, we included two variables from the teacher questionnaire: teacher educational attainment and teacher experience. For the former, teachers were asked “What is the highest level of formal education you have completed?” and were provided seven categories with the lowest value being “Did not complete upper secondary education” and the highest value being Doctorate or equivalent level (IEA, 2019, p.2). For the latter, teachers were asked: “At the end of this school year, how many years will you have taught altogether.” (IEA, 2019, p.2) This measure was used as a numeric variable.

*Educational System Measures.* To examine the association between hunger and national wealth we used gross domestic product (GDP) per capita in current US dollars as a measure of wealth in each country (The World Bank, 2022a). In addition, we used the Gini coefficient (World Bank estimate), which is a measure of income distribution within each system. The coefficient ranges from 0 to 1 with 0 representing perfect equality and 1 representing perfect inequality (The World Bank, 2022b).

The international average of the descriptive statistics for each variable and the correlation between them are located in Table 1. The country-level descriptive statistics are reported in Table 3 Appendix.

**Table 1:** Descriptive Statistics and Correlation Between Variables at the International Level

	Descriptive statistics			Correlation between variables				
	Mean	Median	SD		Achievement	Hunger	SES	Teacher experience
Achievement	478.51	476.60	105.26	Achievement				
Hunger	2.10	2	0.76	Hunger	−0.11			
SES	10.19	10.24	1.70	SES	0.45	− 0.09		
Teacher experience	15.55	14	10.14	Teacher experience	0.08	− 0.03	0.08	
Teacher education	5.21	5	0.73	Teacher education	0.23	− 0.03	0.21	− 0.05

All of the correlations are statistically significant (p<.05)

### Analysis

To address our research aim, we first explored the frequency of hunger and its relationship with GDP per capita and the Gini income inequality index across countries. Then, to examine the relationship between hunger and student achievement, we fitted three multilevel models to the data for each country in the sample, described subsequently (Snijders & Bosker, 2012). We used two-level models in which students are nested within classes. We preferred two-level models since all of the variables we use to answer research questions 2 and 3 are either at the student or class level. The other fundamental reason for using multilevel models is to account for the nested structure of observations. By its sampling design, TIMSS selects students as a whole class, leading to interdependence among students (Martin et al., 2020). Therefore, within-class interdependence violates the traditional linear regression assumptions of independent observations (Weisberg, 2005). As a justification, intraclass correlation (ICC) in TIMSS math scores indicated that more than one-third of the variance in achievement can be attributed to between-class differences on average in 39 countries. The median ICC across countries was 0.37 (See Table 5 Appendix for the ICC across countries).

We begin our analysis with a simpler model, Model 1. In this model, we examine the relationship between hunger and achievement without controlling for student, class, and teacher characteristics. Building on this model, in Model 2, we examine the relationship between hunger and student achievement by controlling for student SES, class average SES, teacher experience, and teacher educational attainment. As a justification, SES, teacher experience, and teacher educational attainment are negatively associated with hunger and positively associated with student achievement (Table 1). Therefore, ignoring these relationships may lead to omitted variable bias. More specifically, if the association between hunger and achievement depends on these covariates, the empirical approach may overestimate the relationship between hunger and achievement since hungry students are more likely to have lower individual and peer socioeconomic status, and less-qualified teachers. Both Model 1 and Model 2 assume fixed relationships between hunger and achievement across classes. Therefore, they are random intercept and fixed slope models. Since the relationship between hunger and achievement may vary across classes, we allow the slopes to vary across classes in Model 3 using the same variables in Model 2. We referred to Model 2 and Model 3 as the “random intercept and fixed slope model”; and the “random intercept and random slope model”, respectively.

Comparing the results from the three multilevel regression models enables us to examine whether and to what extent the relationship between hunger and achievement depends on controlling for student, class, and teacher characteristics. We run the three multilevel regression models for all 39 countries separately. To ease interpretation, we used within-school centering for all student-level predictors except for hunger and grand-mean centering for all class-level predictors (Enders & Tofighi, 2007). We do not center hunger within schools since the mean student achievement is easier to interpret when hunger is “never” rather than the school mean of hunger. Model 2 can be written as follows:

1 $$\begin{gathered} Achievement_{ij} = \,\beta_{0j} \, + \beta_{1} \,Hunger_{ij} \, + \beta_{2} \left( {SES_{ij} } \right)\, + r_{ij} \hfill \\ \hfill \\ \end{gathered}$$

2 $$\beta_{0j} = \gamma_{00} + \gamma_{01} \left( {Class\,SES_{j} } \right)\, + \,\gamma_{02} \left( {TeacherExperience_{j} } \right)\, + \gamma_{03} \left( {TeacherEducation_{j} } \right) + u_{oj}$$

3 $$\beta_{1} = \gamma_{10} ,\beta_{2} = \gamma_{20}$$

Our outcome, ${Achievement}_{ij}$ is TIMSS grade 8 math achievement for student *i* in class *j*. ${Hunger}_{ij}$ is a student-level variable measuring the level of hunger. ${SES}_{ij}$ is student-level socioeconomic status. It is within-class centered by subtracting class means from each student-level observation. ${Class SES}_{j}$ is the grand-mean centered class-level SES, the class mean of student-level SES. ${Teacher Experience}_{j}$ and ${Teacher Education}_{j}$ are the grand-mean centered class-level teacher experience and teacher level of educational attainment, respectively. Among ${\beta }_{0j}$, ${\beta }_{1}$ and ${\beta }_{2}$, only the first parameter is free to vary across classes. ${\beta }_{0j}$ is equal to an overall average achievement value (${\gamma }_{00}$), and effects for class-SES (${\gamma }_{01}$), teacher education (${\gamma }_{02}$), and teacher educational attainment (${\gamma }_{03}$). It has a class-specific error term, (${u}_{0j}$) with variance ${\sigma }_{0}^{2}$.

### Weights, and plausible values

TIMSS, like other international large-scale assessments, uses sampling weights since students and schools do not have the same selection probabilities. Also, estimating the relationship between achievement and variables of interest should consider that all math items are not administered to all students. To reduce the testing burden on individual students and ensure a sufficient number of student responses for each item, TIMSS uses a complex rotated booklet design. Essentially, this creates a missing data structure in which plausible values are used to appropriately estimate achievement levels (Rutkowski et al., 2010). In TIMSS and other large-scale assessments, plausible values refer to random draws from a conditional normal distribution for each student. In TIMSS 2019, there are five plausible values that represent students` math proficiency (Martin et al., 2020).

To address sampling characteristics of TIMSS, we used non-response adjusted student and class weights since multilevel models need to consider weights at both levels. We scaled student weights but not class weights since Level-2 weights do not need to be scaled (Asparouhov, 2006; Nguyen & Kelley, 2018). To appropriately analyze plausible values and combine the results using Rubin`s (1987) multiple imputation approach within a multilevel framework that required student and class weights, we used *mixed.sdf* function in *EdSurvey* that is specifically developed for large-scale assessment studies (Bailey et al., 2021) in R (R Core Team, 2021).

## Results

**Fig. 1:**
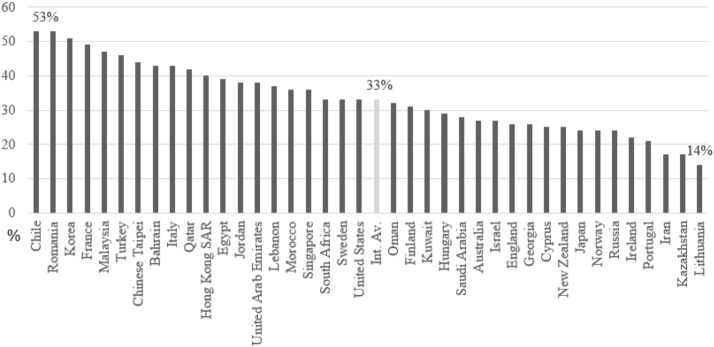
Hunger among students across countries. It is the percentage of grade eight students feel hungry when they arrive at school, every day or almost every day in TIMSS 2019

Results of the prevalence of hunger across countries are shown in Fig. 1. On average, 33% of grade 8 students in TIMSS 2019 reported that they felt hungry every day or almost every day when they arrived at school. In Chile, Romania, and Korea, around half of the students reported hunger. Likewise, with more than 40% of students reporting hunger, France, Malaysia, and Turkey had a higher percentage of hunger than many other countries. On the other hand, the proportion of these students was smaller than 20% in Lithuania, Kazakhstan, and Iran.

**Fig. 2:**
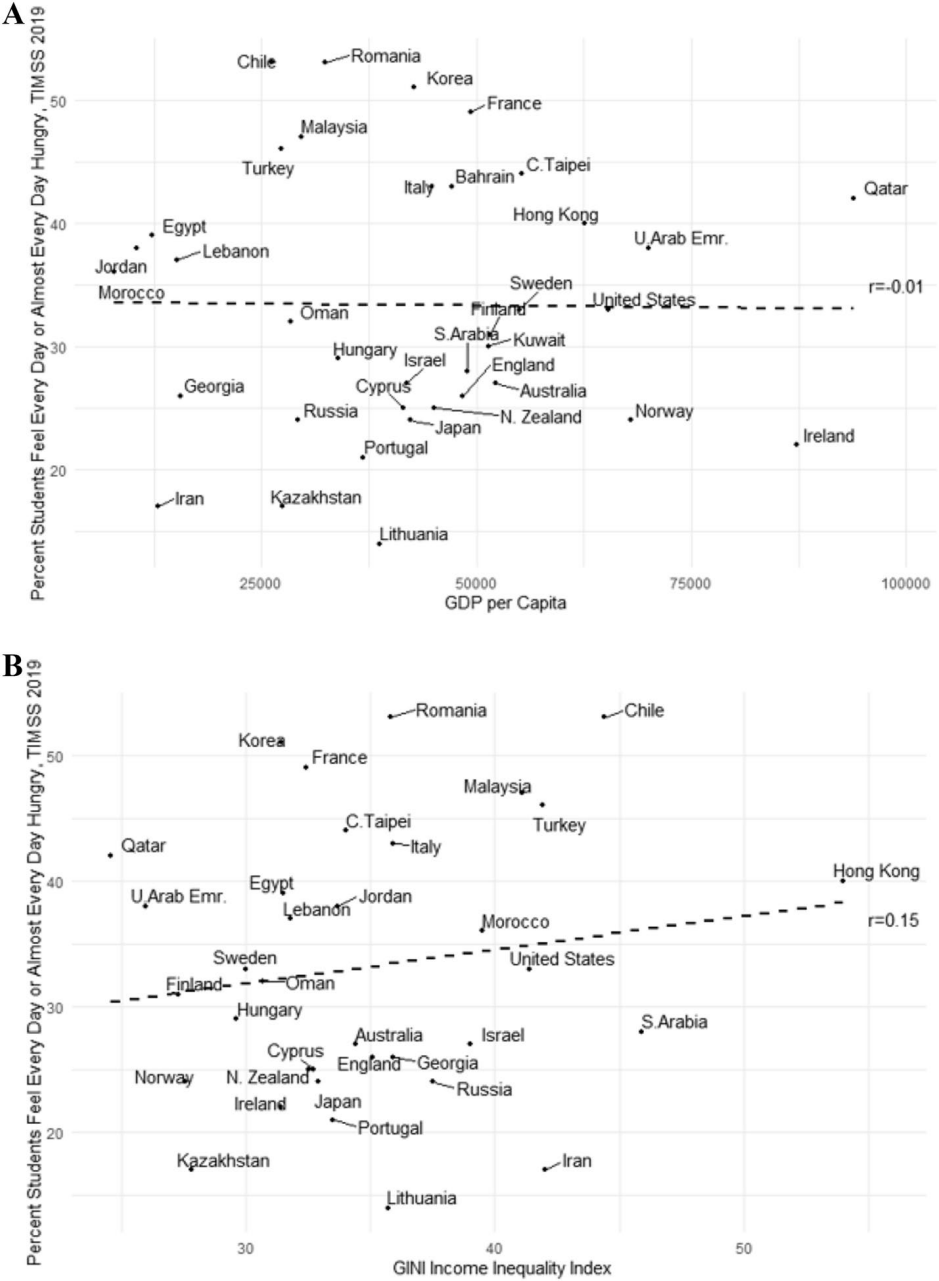
**a**Relationship between GDP per Capita and frequency of hunger among students in TIMSS 2019 across countries. **b**Relationship between income inequalities (i.e., GINI index) and frequency of hunger among students in TIMSS 2019 across countries

Figures 2a, b illustrate the relationship between hunger in TIMSS 2019, GDP per capita, and GINI income inequality across countries. There was no statistically significant relationship between hunger and GDP per capita (*r* (37) = 0.01, *p* > 0.05) as shown in Fig. 2a, corroborating the results in Pereira et al. (2017). There was a moderately high but statistically insignificant relationship between hunger and the GINI coefficient across countries (*r* (35) = 0.15, *p* > 0.05) as illustrated in Fig. 2b. These results suggested that hunger affects students from a large number of countries with diverse economic development around the globe.

Multilevel regression results for the relationship between the frequency of hunger and achievement are reported in Table 2. Model 1 examines the relationship without controlling for student SES, class SES, teacher experience, and teacher educational attainment. It has fixed intercept and slope. Model 2 examines the relationship after controlling for those variables with fixed intercept and slope for all variables. Model 3 examines the relationship after controlling for those variables with fixed intercept and random slope for hunger. To avoid redundancy due to a large number of countries, we only reported the fixed effects in Table 2. Results from the random part (i.e., residual and intercept variance), interclass correlations, R^2^`s, and model fit comparisons are reported in Table 5 Appendix by country. Based on the likelihood-ratio test (χ2), Model 2 fit statistically significantly better than Model 1 in all countries (*df* = 4). There was no model fit difference between Model 2 and Model 3 in almost any country (*df* = 2). Further, the explained variance was higher in Model 2 than Model 1 in all countries. However, there was no substantial difference in explained variance between Model 2 and Model 3 in the majority of the countries (see Table 5 Appendix).

**Table 2:** The Relationship Between Hunger and Achievement (Fixed effects)

Model	Model 1		Model 2						Model 3					
Model explanation	Random intercept and fixed slope model		Random intercept and fixed slope model with control variables						Random intercept and slope model with control variables					
	Intercept	Hunger	Intercept	Hunger	Student SES	Class SES	Teacher experience	Teacher education	Intercept	Hunger	Student SES	Class SES	Teacher experience	Teacher education
Australia	544.99* (4.50)	− 11.68* (1.29)	548.39* (3.57)	− 9.64* (1.25)	6.90* (0.84)	68.18* (3.48)	0.08 (0.22)	− 1.08 (5.11)	549.32* (3.59)	− 10.10* (1.24)	6.93* (0.84)	68.36* (3.48)	0.07 (0.21)	− 1.20 (5.10)
Bahrain	509.01* (5.26)	− 12.14* (1.93)	505.35* (4.83)	− 11.17* (1.90)	5.31* (1.40)	22.61* (3.95)	− 0.41 (0.30)	0.82 (3.14)	505.33* (4.82)	− 11.16* (1.88)	5.30* (1.39)	22.65* (3.95)	− 0.40 (0.29)	0.80 (3.14)
Chile	476.46* (6.72)	− 9.58* (1.65)	472.65* (5.00)	− 8.76* (1.70)	7.31* (1.11)	52.67* (2.84)	0.15 (0.20)	11.70 (7.06)	473.08* (5.0)	− 8.93* (1.69)	7.33* (1.10)	52.63* (2.86)	0.15 (0.19)	11.10 (7.03)
Chinese Taipei	590.21* (10.39)	3.57 (3.95)	607.70* (8.28)	0.73 (3.21)	16.78* (1.18)	47.97* (4.02)	0.61 (0.47)	− 5.86 (6.49)	607.70* (8.28)	0.73 (3.20)	16.78* (1.18)	47.98* (4.02)	0.61 (0.47)	− 5.87 (6.09)
Cyprus	533.60* (5.97)	− 14.12* (2.65)	526.71* (5.05)	− 10.65* (2.53)	17.72* (1.50)	52.14* (3.77)	0.48 (0.35)	8.37 (4.45)	526.60* (5.06)	− 10.60* (2.51)	17.68* (1.48)	52.22* (3.78)	0.46 (0.33)	8.40 (4.38)
Egypt	432.66* (7.46)	− 6.31* (2.29)	434.71* (7.60)	− 7.21* (2.32)	5.88* (0.87)	36.03* (9.67)	0.37 (0.42)	2.08 (5.00)	434.05* (7.20)	− 6.97* (2.16)	5.91* (0.87)	38.24* (8.46)	0.34 (0.40)	1.58 (4.90)
England	553.13* (9.82)	− 16.33* (3.06)	543.41* (6.63)	− 11.85* (2.19)	8.72* (1.44)	62.81* (5.57)	-0.51 (0.55)	− 8.87 (7.70)	543.41* (6.63)	− 11.85* (2.18)	8.72* (1.43)	62.81* (5.56)	− 0.51 (0.54)	− 8.87 (7.70)
Finland	547.70* (5.44)	− 18.94* (2.23)	540.72 (12.77)	− 18.31* (5.04)	13.41* (4.30)	51.11* (8.97)	0.87 (0.65)	0.14 (3.21)	541.7* (4.60)	− 15.82* (2.01)	14.12* (1.47)	45.78* (3.32)	0.33 (0.17)	4.92 (3.75)
France	515.66* (5.28)	− 11.53* (1.83)	506.56 (16.13)	− 6.97* (6.53)	16.52* (2.38)	50.89* (5.43)	− 0.52 (0.58)	6.64 (3.18)	505.60* (4.14)	− 7.48* (1.55)	15.54* (1.08)	42.44* (2.82)	0.12 (0.17)	3.59 (2.68)
Georgia	477.55* (6.20)	− 6.11* (2.23)	477.38* (5.47)	− 5.84* (2.15)	11.73* (1.36)	35.24* (7.02)	− 0.55 (0.35)	7.76 (5.97)	477.31* (5.46)	− 5.81* (2.15)	11.73* (1.36)	35.24* (7.01)	− 0.57 (0.36)	8.04 (5.97)
Hong Kong SAR	597.83* (8.75)	− 10.25* (2.67)	598.82* (7.26)	− 8.60* (2.52)	2.68^+^ (1.43)	53.82* (5.20)	0.07 (0.42)	− 2.93 (7.85)	598.67* (7.21)	− 8.52* (2.49)	2.68 (1.42)	53.84* (5.20)	0.07 (0.42)	− 2.98 (7.83)
Hungary	545.17* (5.20)	− 12.06* (1.74)	548.10* (3.72)	− 10.25* (1.64)	16.86* (1.03)	54.54* (2.30)	0.46* (0.21)	12.47* (3.59)	547.72* (3.73)	− 10.10* (1.65)	16.86* (1.03)	54.48* (2.31)	0.47* (0.20)	12.29* (3.61)
Iran, Islamic rep. of	470.15* (5.11)	− 11.38* (1.84)	464.89* (4.03)	− 9.08* (1.80)	8.31* (0.93)	44.66* (3.07)	− 0.08 (0.30)	0.06 (3.83)	465.00* (3.98)	− 9.14* (1.77)	8.34* (0.93)	44.65* (3.07)	− 0.08 (0.30)	0.03 (3.82)
Ireland	555.76* (4.74)	− 16.08* (1.96)	548.02* (3.71)	− 10.29* (1.67)	16.25* (0.94)	41.53* (2.83)	0.19 (0.16)	− 0.20 (3.48)	548.02* (3.71)	− 10.29* (1.69)	16.25* (0.94)	41.53* (2.83)	0.19 (0.16)	− 0.20 (3.48)
Israel	547.32* (9.04)	− 10.18* (3.30)	543.09* (7.48)	− 8.57* (2.82)	11.68* (1.38)	54.93* (4.69)	1.49* (0.42)	1.37 (5.15)	543.01* (7.46)	− 8.55* (2.81)	11.68* (1.37)	54.91* (4.69)	1.50* (0.42)	1.41 (5.16)
Italy	530.20* (5.16)	− 13.11* (2.01)	522.57* (4.45)	− 9.74* (1.71)	12.87* (0.90)	28.83* (3.07)	− 0.07 (0.18)	− 3.30 (5.18)	522.57* (4.45)	-9.74* (1.71)	12.87* (0.90)	28.83* (3.07)	− 0.07 (0.18)	− 3.30 (5.18)
Japan	620.66* (5.27)	− 14.71* (2.26)	622.92* (4.91)	− 14.31* (2.12)	16.95* (1.27)	63.22* (10.29)	0.25 (0.19)	− 5.63 (4.02)	622.92* (4.91)	− 14.31* (2.12)	16.95* (1.27)	63.22* (10.29)	0.25 (0.19)	− 5.62 (4.02)
Jordan	443.62* (5.24)	− 11.05* (1.90)	441.28* (4.81)	− 10.07* (1.84)	6.04* (0.95)	34.91* (5.33)	0.47 (0.31)	1.50 (3.88)	441.86 (4.87)	− 10.34* (1.86)	6.01* (0.95)	34.88* (5.32)	0.48 (0.31)	1.74 (3.85)
Kazakhstan	490.42* (5.64)	0.15 (2.03)	489.20* (5.22)	0.96 (1.94)	6.20* (1.57)	39.80* (5.07)	0.30 (0.27)	7.43 (7.34)	489.20* (5.25)	0.97 (1.95)	6.20* (1.57)	39.80* (5.07)	0.30 (0.27)	7.44 (7.33)
Korea, Rep. of	624.49* (7.75)	− 11.02* (3.19)	623.04* (7.34)	− 8.40* (3.08)	20.17* (1.12)	49.32* (5.22)	-0.02 (0.24)	− 1.45 (4.73)	623.04* (7.34)	− 8.40* (3.08)	20.17* (1.12)	49.32* (5.22)	− 0.02 (0.24)	− 1.45 (4.73)
Kuwait	426.77* (5.67)	− 10.81* (2.33)	426.99* (5.18)	− 10.69* (2.40)	4.95* (1.25)	47.51* (12.58)	− 0.12 (0.55)	18.73 (12.25)	427.64* (5.20)	− 11.02* (2.34)	4.96* (1.25)	47.36* (12.49)	− 0.13 (0.55)	18.80 (12.13)
Lebanon	437.93* (5.34)	− 4.96* (1.73)	440.03* (4.59)	− 5.33* (1.72)	2.90* (0.69)	35.27* (3.50)	0.49 (0.25)	2.50 (2.15)	439.99* (4.59)	− 5.32* (1.72)	2.90* (0.69)	35.28* (3.47)	0.49* (0.25)	2.46 (2.16)
Lithuania	522.70* (5.39)	− 4.48* (2.13)	518.48* (4.07)	− 0.78 (2.00)	21.28* (1.34)	56.06* (3.79)	0.52* (0.22)	9.34* (3.91)	518.48* (4.06)	− 0.78 (1.99)	21.28* (1.33)	56.04* (3.80)	0.52* (0.22)	9.35* (3.92)
Malaysia	489.77* (6.68)	− 1.37 (1.66)	496.98* (4.98)	− 3.43* (1.62)	4.46* (0.68)	73.19* (3.09)	− 0.31 (0.37)	2.08 (6.43)	496.67* (5.00)	− 3.31* (1.63)	4.45* (0.68)	78.14* (3.09)	− 0.31 (0.37)	2.06 (6.45)
Morocco	401.75* (5.79)	− 5.08* (1.97)	400.10* (5.06)	− 5.49* (1.92)	2.97* (0.75)	20.46* (3.39)	0.37 (0.27)	4.51 (2.34)	400.11* (5.04)	− 5.50* (1.90)	2.97* (0.75)	20.47* (3.39)	0.37 (0.27)	4.53 (2.34)
New Zealand	519.00* (5.73)	− 13.73* (1.84)	519.22* (4.34)	− 10.10* (1.78)	10.70* (0.93)	71.58* (3.33)	0.11 (0.22)	8.20 (4.05)	519.11* (4.32)	− 10.05* (1.77)	10.70* (0.93)	71.54* (3.32)	0.15 (0.22)	8.21 (4.05)
Norway	538.43* (6.00)	− 16.11* (2.21)	533.01* (4.61)	− 13.14* (1.97)	17.76* (1.26)	34.17* (4.34)	− 0.28 (0.23)	2.33 (4.24)	533.01* (4.60)	− 13.15* (1.97)	17.77* (1.26)	34.15* (4.34)	− 0.28 (0.23)	2.33 (4.25)
Oman	446.30* (6.72)	− 13.23* (2.61)	446.50* (5.77)	− 11.82* (2.41)	13.15* (1.04)	49.69* (3.74)	− 0.63 (0.49)	20.92* (5.58)	446.43* (5.75)	− 11.78* (2.39)	13.14* (1.04)	49.65* (3.74)	− 0.64 (0.49)	20.95* (5.58)
Portugal	537.84* (5.96)	− 17.49* (2.48)	528.36* (4.76)	− 13.18* (2.19)	11.63* (1.02)	34.87* (2.63)	0.26 (0.30)	8.96* (4.55)	528.36* (4.76)	− 13.17* (2.19)	11.63* (1.03)	34.87* (2.63)	0.26 (0.30)	8.96* (4.56)
Qatar	494.05* (6.69)	− 17.88* (2.05)	485.58* (5.72)	− 14.30* (2.07)	11.29* (1.13)	62.64* (6.77)	− 0.44 (0.42)	18.55* (6.83)	485.59* (5.71)	− 14.30* (2.07)	11.29* (1.13)	62.65* (6.77)	− 0.45 (0.42)	18.56* (6.83)
Romania	525.05* (7.55)	− 14.35* (2.62)	519.41* (6.55)	− 11.15* (2.50)	16.90* (1.31)	49.60* (3.22)	0.23 (0.28)	0.53 (4.21)	519.35* (6.52)	− 11.13* (2.49)	16.88* (1.31)	49.64* (3.23)	0.24 (0.28)	0.56 (4.22)
Russian Federation	545.00* (5.44)	− 0.65 (2.00)	550.12* (4.87)	− 1.52 (1.99)	8.82* (1.23)	42.65* (5.94)	− 0.25 (0.23)	5.36 (6.10)	550.19* (4.86)	− 1.56 (1.99)	8.82* (1.23)	42.58* (5.95)	− 0.25 (0.23)	5.44 (6.10)
Saudi Arabia	418.51* (5.19)	− 6.11* (1.80)	417.23* (4.64)	− 5.65* (1.75)	7.06* (1.31)	51.37* (4.87)	0.28 (0.44)	16.59 (19.81)	417.19* (4.64)	− 5.65* (1.76)	7.06* (1.31)	51.31* (4.87)	0.28 (0.44)	16.33 (19.66)
Singapore	634.36* (5.39)	− 9.86* (1.24)	629.94* (3.81)	− 8.90* (1.25)	0.79 (0.67)	82.90* (3.64)	− 0.43 (0.33)	− 6.82 (5.08)	630.01* (3.82)	− 8.92* (1.25)	0.79 (0.67)	82.89* (3.64)	− 0.43 (0.33)	− 6.81 (5.08)
South Africa	423.29* (3.87)	− 7.58* (0.99)	415.35* (2.85)	− 5.65* (0.91)	0.56 (0.46)	55.27* (2.24)	0.08 (0.18)	6.63* (3.27)	415.82* (2.77)	− 5.86* (0.94)	0.56 (0.46)	55.28* (2.23)	0.086 (0.18)	6.66* (3.26)
Sweden	536.61* (6.11)	− 14.06* (2.38)	527.07* (4.24)	− 8.87* (1.92)	18.23* (1.06)	46.54* (3.20)	0.16 (0.19)	0.92 (1.68)	527.06* (4.24)	− 8.87* (1.92)	18.23* (1.06)	46.54* (3.20)	0.16 (0.20)	0.92 (1.68)
Turkey	470.39* (9.26)	8.04* (3.21)	491.77* (8.43)	1.22 (3.23)	14.21* (1.71)	42.53* (3.73)	− 0.58 (0.51)	15.90* (6.83)	490.91* 8.61	1.14 (3.30)	14.69* (1.74)	42.55* (3.90)	− 0.63 (0.51)	15.94* (6.83)
United Arab Emirates	494.99* (3.63)	− 10.48* (0.93)	490.70* (3.02)	− 8.94* (0.93)	5.46* (0.53)	59.83* (2.94)	− 1.40* (0.25)	14.44* (3.16)	490.68* (3.03)	− 8.93* (0.93)	5.46* (0.53)	59.80* (2.94)	− 1.39* (0.25)	14.45* (3.15)
United States	549.86* (6.10)	− 15.87* (2.02)	533.30* (4.69)	− 8.01* (1.85)	6.96* (0.91)	61.49* (2.76)	0.06 (0.31)	6.05 (5.0)	530.66* (6.56)	− 8.72* (2.78)	7.39* (1.27)	54.78* (4.17)	0.37 (0.40)	10.98* (3.54)

Significance codes: *p < 0.05

In Model 1, there was a statistically significant and negative relationship between hunger and student achievement in most countries. The magnitude of the estimated negative hunger coefficient ranged from about 5% to 19% of a standard deviation on the TIMSS scale. For instance, at the extreme, one unit increase in hunger was associated with lower math achievement by − 18.94, − 17.88, and − 17.49 points in Finland, Qatar, and Portugal, respectively. In approximately two-thirds of the countries (*N* = 25), the negative relationship was larger than 10% of a standard deviation on the TIMSS scale. Among 39 countries, only five countries did not have a statistically significant relationship between hunger and student achievement.

In Model 2, we found that the relationship between hunger and achievement did not change substantially once student SES, class SES, teacher experience, and teacher educational attainment were taken into account. In many countries, the negative relationship between hunger and student achievement reduced slightly and remained statistically significant after controlling for those variables. For instance, the coefficients of hunger in Model 1 and Model 2 were − 11.68 and − 9.64 in Australia; − 12.06 and − 10.25 in Hungary; and − 6.11 and − 5.84 in Georgia, respectively. These results suggested that hunger has a unique relationship with student achievement independent of the student, class, and teacher characteristics.

In some other countries, however, there were relatively larger changes between Model 1 and Model 2. For instance, the coefficient of frequency of hunger dropped from − 15.87 to − 8.01 in the US, from − 16.33 to − 11.85 in England; from − 14.06 to − 8.87 in Sweden once student, class, and teacher characteristics were included. However, the coefficients remained statistically significant. A relatively larger change in the coefficient of hunger between Model 1 and Model 2 appeared to be the result of a stronger correlation between hunger and student SES in these countries. For instance, the US has the strongest relationship between hunger and SES among 39 countries, suggesting that there was a relatively larger gap in access to sufficient food between socioeconomically advantaged and disadvantaged students. Likewise, Sweden and England have relatively larger relationships between hunger and SES (see Table 3 Appendix). Despite a relatively larger change in the magnitude of the relationship, once control variables are considered, the negative relationships between hunger and achievement were still statistically significant in these three countries in both models.

Overall, Model 2 suggested that even controlling for student SES, class SES, and teacher characteristics, there is a negative and statistically significant relationship between hunger and achievement in 34 of 39 countries ranging from − 3.43 in Malaysia to − 18.31 in Finland. Equivalently, these results suggest, controlling for other variables, a one-unit increase in the hunger scale was expected to decrease math achievement from about 3–18% of a standard deviation on the TIMSS scale across countries. Put differently, controlling for other variables, the achievement gap between students who never come to school hungry and those who always or almost always come to school hungry ranges from 6 to 36% of a standard deviation on the TIMSS scale across countries.

Model 3 indicated that allowing the relationship between hunger and student achievement to vary across classes did not change the results compared to Model 2. In all countries, the magnitude of the relationship between hunger and achievement was quite stable between Model 2 and Model 3. In a few countries, the magnitude of the negative relationship changed only marginally between Model 2 and Model 3. For instance, at the extreme, the magnitude of the relationship increased from − 8.01 to − 8.72 in the US whereas it decreased from − 18.31 to − 15.31 in Finland. Further, only Chinese Taipei, Kazakhstan, Russian Federation, and Turkey had no statistically significant relationships between hunger and student achievement in any of the three multilevel regression models. Finally, Model 2 and Model 3 indicated that student and class SES are significantly and strongly associated with student achievement as expected. Controlling for other variables, teacher experiences and teacher education were not significant predictors of student achievement in most countries. These results do not necessarily imply an insignificant relationship between teacher experiences, teacher education, and student achievement. As reported in Table 1, there is a positive relationship between these teacher characteristics and student achievement. Yet, the multilevel regression results suggest the relationship of student achievement with student hunger as well as its relationship with student and class socioeconomic suppress the relationship between teacher characteristics and student achievement.

## Discussion

Internationally, access to food has historically been a conversation focused towards low-income countries (Alderman et al., 2006). However, a growing body of research reveals that a significant number of households from high, and middle-income countries lack adequate nutrition (Pereira et al., 2017; Pollard & Booth, 2019). Indeed, our findings confirm that students from around the world go to school hungry. Specifically, across the 39 educational systems in TIMSS, we found that about one in three students arrived at school hungry and that the proportion of food-insecure students was independent of the countries’ economic prosperity. What is unique about our findings is that students were specifically asked to report their own experiences, which differs from many other large-scale studies that normally depend on household surveys completed by adults. With this unique international perspective, we found that students who were food-insecure had lower math achievement than their peers. Though the magnitude of the achievement gap by the frequency of hunger ranges across countries, there is a consistent, negative relationship between hunger and achievement. Further, we found that even after controlling for several background variables, food-insecure students had lower achievement than their food-secure peers. These results implied that unequal access to sufficient food intensifies the achievement gap between wealthier and disadvantaged students. Thus, our findings confirm the results from national, regional, and local studies (e.g., Kim et al., 2003; Lien, 2007; Metwally et al., 2020; Taha & Rashed, 2017), demonstrating that hunger is a global issue.

The global relationship between hunger and achievement raises important questions about what can and should be done to ensure students have access to food. For example, many countries have some type of program that provides food for needy students. Internationally, one out of every two children, or 388 million, receive daily school meals and in most high-income countries the coverage rate is more than three out of four (WFP, 2020). Despite the wide reach of school meal programs, a logical question is why do we observe so many students that are hungry? The underlying reasons may be numerous, and future research is certainly needed, but some ideas are worth discussing. First, school meals may only partially avert the negative effect of hunger on learning and achievement. Having nutritious meals at school can help boost student achievement through improved attention and motivation at school; however, most students do not fully board at their school and must rely on their home environment for most of their nutritional needs. Therefore, school meal programs may have some limitations to mitigate the disadvantages associated with hunger, especially on weekends and during school holidays. Second, perceived social stigma prevents some students from participating in meal programs even if they are eligible to eat school meals (Dotter, 2013; Schwartz & Rothbart, 2020). An author of this paper recalls having to stand in separate lines to receive free lunches at school, leading many of the students who qualified for free meals to skip eating rather than deal with the social stigma brought on by receiving perceived handouts. Although personal experiences are not generalizable to an international context, it is reasonable to assume that in many societies receiving food subsidies comes with a negative social stigma.

Further, even if students receive one meal at school, TIMSS data captured the level of hunger when students arrived at school, which is largely a measure of missing breakfast or lacking a sufficient breakfast rather than lunch or dinner. In fact, in many countries, free meal programs are solely focused on lunch (WFP, 2020). Even in countries that offer free breakfast, the amount of children who receive meals is much lower when compared to those who receive lunch. For instance, in the US, only half of the students who participate in the national lunch program participate in the school breakfast program (USDA, 2020). The Survey of School Meal Programs indicates that the majority of large-scale school meal programs across the globe offer lunch (%90) but fewer programs offer breakfast (40%) (Global Child Nutrition Foundation, 2022). A clear policy recommendation from our results suggests that all countries that participated in TIMSS need to examine their school meal programs and explore ways to feed the students who show up to school hungry. When students must wait until lunch to satisfy their hunger, important instructional time is lost. In fact, since most of the school day in many countries falls between breakfast and lunch, ensuring students have food in the morning is imperative.

## Conclusion

Given that research shows that hunger is associated with increased behavioral issues, the problems associated with having hungry students in the classroom move beyond the simple influence on achievement. In fact, how can societies possibly expect students whose basic human needs are not being met to participate and fully engage in school? Having hungry students in our schools is a violation of basic human rights. Emerging programs that show promising results include comprehensive school meal programs integrated with the social welfare and protection programs (see WFP, 2020; Sandefur, 2022). Moreover, providing meals at schools has been shown to improve learning and combat inequalities, especially in low-and middle-income contexts (Bedasso, 2022). Several cost–benefit analysis studies investigated whether and to what extent school meal programs yield a return on investment (e.g., Chakraborty & Jayaraman, 2019). For instance, a meta-analysis ranked school meal programs as the third most effective policy alternative to boost student learning, after structured pedagogy and extra time (Bashir et al., 2018). School meal programs are also among the most effective intervention programs to improve learning-adjusted years of schooling (Angrist et al., 2020). Despite the limitations to uncovering causal mechanisms, our findings suggest that the relationship between hunger and achievement is substantial and stable within and across countries. We estimated that controlling for other variables, the achievement gap between students who never come to school hungry and those who come to school always or almost always hungry is significant and deserves our attention.

Although we look at the association between hunger and achievement in this paper, achievement gaps are an artifact of denying children a basic human right. While understanding the effectiveness of policy alternatives relative to school meal programs may provide important information about choosing among policy alternatives, the underlying logic of such an approach ignores that by its nature, hunger resulting from food insecurity is a failure of modern society. Further, aside from moral reasons and additional benefits such as social protection and improved income, health, and school participation (Aurino et al., 2020; Bedasso, 2022; Imberman & Kugler, 2012; Lundborg et al., 2022; Schwartz & Rothbart, 2020), hunger has another crucial role: It may inhibit the success of other policy alternatives. As suggested by Maslow’s theory, if students are hungry, there is little schools can do to ensure higher-level learning.

Knowledge about how hunger interacts with and mediates other policy instruments is limited. This is in part because most studies have focused on the relationship between hunger and achievement and the effect of school meal programs. For instance, little is known about how hunger influences the learning process and classroom environment individually and ecologically. Future studies can examine those issues to provide more empirical evidence around whether hunger is a pre-request for other policy options. This can provide more nuanced evidence about how hunger imposes barriers to ensuring equal learning opportunities for vulnerable students across a wide range of social and economic spectra around the globe.

Like other studies that use international assessment data, our study has a number of limitations. First, the cross-sectional nature of the data prevents us from isolating hunger as a cause of achievement. Second, we do not know why the students come to school hungry. For example, although hunger is largely associated with lower SES, in some cultures, it may be that breakfast is not a normal part of the local diet. That said, given the overwhelming research that suggests that hungry students have a difficult time learning even the reasons for coming to school hungry are less important than the fact that students are hungry. Our study is also limited by the extent to which the variables and underlying constructs used in this study function in the same way across systems. That is, language and cultural differences can result in different conceptualizations or interpretations across educational systems. In addition, there is potentially a social desirability bias in self-reported hunger measures. Given that social desirability is culturally shaped (Keillor et al., 2001), the intersection of social desirability and cross-cultural comparisons poses additional limitations. Even though addressing social desirability bias and detecting its magnitude are challenging, the negative relationship between the frequency of student hunger and socioeconomic status which is consistent across most countries suggests that the social desirability bias in hunger measure is likely not substantial. In other words, consistent with expectations, socioeconomically disadvantaged students report higher levels of hunger since they have fewer resources to access nutritious food. Finally, we did not detect a significant relationship between hunger and student achievement in four countries. Our dataset did not allow us to unpack the underlying reason for the null relationship in those countries. Future studies can further examine this issue by exploiting national or regional datasets. Despite these limitations, our findings provide important insights and demonstrate that all countries need to do more to ensure all of their children’s basic needs are met.

## Data Availability

TIMSS 2019 data used in this study is publicly available on IEA`s website https://timss2019.org/international-database/.

## Appendix

See Tables 3, 4, 5

**Table 3:** Sample Size and Descriptive Statistics by Country

Country	Sample Size	Achievement		Hunger		SES		Teacher experience		Teacher education		Correlation between hunger and SES
	N	Mean	SE	Mean	SE	Mean	SE	Mean	SE	Mean	SE	
Australia	9060	517.28	3.8	1.97	0.01	11.21	0.02	13.94	0.12	5.22	0.00	− 0.12
Bahrain	5725	481.09	1.7	2.22	0.01	10.24	0.02	13.94	0.11	5.17	0.01	− 0.08
Chile	4115	440.61	2.8	2.39	0.01	10.00	0.02	14.64	0.19	5.10	0.01	− 0.05
Chinese Taipei	4915	612.50	2.7	2.36	0.01	10.35	0.02	17.00	0.11	5.60	0.01	0.00
Cyprus	3521	501.08	1.6	1.89	0.01	10.92	0.02	12.10	0.15	5.68	0.01	−0.09
Egypt	7210	412.88	5.2	2.20	0.01	9.46	0.02	18.52	0.12	4.67	0.01	0.00
England	3365	514.93	5.3	1.96	0.01	10.73	0.03	12.73	0.14	5.33	0.01	− 0.11
Finland	4874	508.92	2.6	2.13	0.01	11.16	0.02	14.37	0.13	5.94	0.01	− 0.09
France	3874	482.61	2.5	2.32	0.01	10.66	0.02	14.74	0.14	5.76	0.01	− 0.10
Georgia	3315	461.30	4.3	1.96	0.01	10.90	0.03	24.70	0.20	5.82	0.01	− 0.02
Hong Kong	3265	578.31	4.1	2.28	0.01	10.24	0.03	14.97	0.17	5.44	0.01	− 0.04
Hungary	4569	516.54	2.9	2.01	0.01	11.11	0.02	25.51	0.15	5.36	0.01	− 0.08
Iran	5980	446.17	3.7	1.75	0.01	9.50	0.02	17.67	0.11	5.23	0.01	− 0.11
Ireland	4118	523.73	2.6	1.89	0.01	10.86	0.02	13.95	0.11	5.43	0.01	− 0.12
Israel	3731	519.11	4.3	1.98	0.01	11.02	0.02	15.53	0.13	5.45	0.01	− 0.07
Italy	3619	497.48	2.7	2.21	0.01	10.38	0.03	19.57	0.18	6.08	0.01	− 0.09
Japan	4446	594.23	2.7	1.89	0.01	10.87	0.02	15.46	0.14	5.12	0.00	− 0.03
Kazakhstan	4453	487.56	3.3	1.87	0.01	10.26	0.02	19.89	0.18	5.11	0.01	− 0.05
Jordan	7176	420.27	4.3	2.14	0.01	9.52	0.02	11.04	0.10	4.74	0.01	− 0.06
Korea	3861	606.82	2.8	2.40	0.01	11.60	0.02	15.71	0.15	5.37	0.01	− 0.07
Kuwait	4574	402.75	5.0	2.06	0.01	9.87	0.02	13.60	0.11	5.12	0.01	− 0.04
Lebanon	4730	429.31	2.9	2.13	0.01	9.66	0.02	13.56	0.14	5.14	0.01	0.00
Lithuania	3826	520.43	2.9	1.69	0.01	10.81	0.02	28.74	0.15	5.50	0.01	− 0.06
Malaysia	7065	460.57	3.2	2.41	0.01	9.86	0.02	13.80	0.09	5.02	0.01	0.05
Morocco	8458	388.19	2.3	2.17	0.01	8.25	0.02	12.25	0.13	3.95	0.02	− 0.03
New Zealand	6051	481.59	3.4	1.91	0.01	10.98	0.02	16.36	0.13	5.52	0.01	− 0.15
Norway	4575	502.87	2.4	1.93	0.01	11.42	0.02	12.24	0.14	5.47	0.01	− 0.08
Oman	6751	410.66	2.8	2.07	0.01	9.73	0.02	13.99	0.08	5.06	0.01	− 0.07
Portugal	3377	500.32	3.2	1.73	0.01	10.36	0.03	20.88	0.12	5.19	0.01	− 0.12
Qatar	3884	443.42	4	2.21	0.01	10.39	0.02	12.68	0.14	5.27	0.01	− 0.07
Romania	4494	478.99	4.3	2.40	0.01	10.51	0.02	23.88	0.15	5.15	0.01	− 0.07
Russian Federation	3901	543.49	4.5	1.92	0.01	10.64	0.02	25.54	0.19	5.74	0.01	− 0.00
Saudi Arabia	5680	393.77	2.5	2.00	0.01	9.59	0.02	11.61	0.09	5.03	0.00	− 0.04
Singapore	4853	615.77	4	2.18	0.01	10.50	0.02	11.16	0.12	5.17	0.01	− 0.14
South Africa	20829	389.48	2.3	2.13	0.01	9.10	0.01	14.09	0.07	4.78	0.00	− 0.09
Sweden	3996	502.52	2.5	2.08	0.01	11.03	0.02	15.75	0.14	5.23	0.02	− 0.13
Turkey	4077	495.63	4.3	2.35	0.01	9.38	0.03	10.46	0.10	5.06	0.00	0.09
United Arab Emirates	22334	473.43	1.9	2.16	0.01	10.33	0.01	13.69	0.05	5.27	0.00	− 0.11
United States	8698	515.44	4.8	2.06	0.01	10.66	0.02	14.48	0.10	5.57	0.01	− 0.17

**Table 4:** Correlation between Frequency of Hunger and Student Achievement with and without aggregating “Every Day” and “Almost Every Day” Response Categories

Country	Every day and almost every day aggregated	Every day and almost every day not aggregated	Country	Every day and almost every day aggregated	Every day and almost every day not aggregated
Australia	−0.19	− 0.20	Lithuania	− 0.03	− 0.03
Bahrain	− 0.10	− 0.11	Malaysia	0.02	0.00
Chile	− 0.09	− 0.10	Morocco	− 0.06	− 0.06
Chinese Taipei	0.00	− 0.01	Oman	− 0.11	− 0.13
Cyprus	− 0.14	− 0.14	New Zealand	− 0.20	− 0.22
Finland	− 0.19	− 0.20	Norway	− 0.14	− 0.14
France	− 0.13	− 0.13	Portugal	− 0.19	− 0.18
Georgia	− 0.06	− 0.06	Qatar	− 0.19	− 0.23
Hong Kong	− 0.10	− 0.11	Romania	− 0.12	− 0.12
Hungary	− 0.14	− 0.14	Russian federation	0.00	0.00
Iran	− 0.12	− 0.12	Saudi Arabia	− 0.04	− 0.05
Ireland	− 0.18	− 0.19	South Africa	− 0.09	− 0.10
Israel	− 0.10	− 0.10	Sweden	− 0.16	− 0.17
Italy	− 0.15	− 0.15	United Arab Emirates	− 0.18	− 0.19
Japan	− 0.14	− 0.14	Turkey	0.08	0.07
Kazakhstan	− 0.01	− 0.02	Egypt	− 0.05	− 0.05
Jordan	− 0.11	− 0.10	United States	− 0.17	− 0.19
Korea	− 0.09	− 0.10	England	− 0.15	− 0.16
Kuwait	− 0.11	− 0.13	International average	− 0.11	− 0.11
Lebanon	− 0.05	− 0.06			

**Table 5:** The Relationship Between Hunger and Achievement: Random Effects, Model Summary, and Model Fit

Country	Model 1					Model 2					Model 3						Model comparison			
	Random effects (variance)		ICC	R^2^	− 2LL	Random effects (variance)		ICC	R^2^	− 2LL	Random effects (variance)			ICC	R^2^	− 2LL	Model 1 and model 2		Model 2 and model 3	
	Intercept	Residual				Intercept	Residual				Intercept	Residual	Hunger				χ2	p	χ2	p
Australia	4661	3106	0.60	0.01	90897	2047	3004	0.41	0.34	90322	1860	2998	10	0.41	0.35	90320	575.01	< 0.05	2.23	> 0.05
Bahrain	888.6	8078	0.10	0.01	63270	669	8008	0.08	0.04	63179	894	8007	3	0.08	0.05	63178	90.77	< 0.05	0.90	> 0.05
Chile	2768	3253	0.46	0.01	40960	552	3185	0.15	0.36	40662	1021	3179	13	0.15	0.39	40656	298.43	< 0.05	5.37	> 0.05
Chinese Taipei	2153	7456	0.22	0.00	57259	757	6828	0.10	0.21	56673	1510	6767	165	0.11	0.21	56670	585.68	< 0.05	3.47	> 0.05
Cyprus	1493	5042	0.23	0.01	27291	406	4526	0.08	0.26	26917	323	4524	4	0.08	0.26	26917	373.74	< 0.05	0.32	> 0.05
Egypt	3133	5251	0.37	0.00	66005	2595	5158	0.34	0.08	65873	3780	5099	123	0.34	0.08	65860	131.51	< 0.05	12.28	< 0.05
England	4474	3312	0.58	0.01	24053	1894	3153	0.38	0.35	23858	2022	3138	27	0.38	0.35	23858	195.12	< 0.05	0.30	> 0.05
Finland	1788	3673	0.33	0.02	53219	788	3389	0.19	0.22	52628	712	3379	21	0.19	0.22	52627	591.08	< 0.05	0.90	> 0.05
France	1086	3463	0.24	0.01	32032	221	3026	0.07	0.29	31486	272	3024	1	0.07	0.30	31486	545.97	< 0.05	0.14	> 0.05
Georgia	2776	4883	0.36	0.00	33865	2047	4655	0.31	0.12	33682	1780	4652	3	0.31	0.16	33682	182.43	< 0.05	0.64	> 0.05
Hong Kong	4611	3312	0.58	0.00	33641	2429	330	0.42	0.27	33550	2597	3305	1	0.39	0.27	33549	91.70	< 0.05	0.19	> 0.05
Hungary	3035	4341	0.41	0.01	49460	511	3889	0.12	0.41	48640	627	3878	18	0.12	0.41	48640	819.92	< 0.05	0.25	> 0.05
Iran	3102	4849	0.39	0.01	66142	891	4703	0.16	0.30	65725	746	4698	9	0.16	0.30	65723	417.42	< 0.05	1.41	> 0.05
Ireland	1037	3822	0.21	0.02	42881	238	3360	0.07	0.27	42203	342	3330	58	0.07	0.27	42200	677.19	< 0.05	3.73	> 0.05
Israel	5162	4516	0.53	0.00	30438	2084	4311	0.33	0.32	30187	2942	4300	19	0.33	0.32	30182	250.51	< 0.05	5.30	> 0.05
Italy	1004	3931	0.20	0.02	39353	541	3629	0.13	0.17	38984	659	3610	33	0.13	0.17	38983	369.16	< 0.05	0.67	> 0.05
Japan	828.2	5989	0.12	0.02	50109	362	5447	0.06	0.17	49618	160	5434	21	0.06	0.17	49614	490.35	< 0.05	4.59	> 0.05
Jordan	1947	4757	0.29	0.01	65664	1436	4668	0.24	0.10	65500	1097	4665	4	0.24	0.10	65497	164.20	< 0.05	2.86	> /05
Kazakhstan	2993	3863	0.44	0.44	45233	2295	3813	0.38	0.11	45128	2908	3786	61	0.38	0.11	45125	104.34	< 0.05	3.77	> 0.05
Korea	969.5	7983	0.11	0.01	45229	308	7276	0.04	0.16	44763	145	7271	13	0.04	0.16	44762	465.70	< 0.05	0.92	> 0.05
Kuwait	2442	4619	0.35	0.01	44049	2015	4566	0.31	0.08	43975	1457	4562	10	0.31	0.08	43970	74.70	< 0.05	4.17	> 0.05
Lebanon	1871	2719	0.41	0.01	45062	944	2697	0.26	0.20	44902	1112	2668	54	0.27	0.20	44897	160.30	< 0.05	4.58	> 0.05
Lithuania	2294	4684	0.33	0.00	39859	706	4210	0.14	0.29	39291	668	4209	1	0.14	0.29	39291	568.12	< 0.05	0.04	> 0.05
Malaysia	6199	2605	0.70	0.01	75286	1920	2577	0.43	0.48	74877	2540	2543	88	0.44	0.48	74866	408.64	< 0.05	10.98	< 0.05
Morocco	1280	3437	0.27	0.00	66387	802	3421	0.19	0.10	66274	985	3404	35	0.19	0.10	66272	113.13	< 0.05	1.76	> 0.05
New Zealand	4725	3920	0.55	0.01	61736	1358	3723	0.27	0.41	61165	1269	3692	61	0.27	0.41	61159	570.76	< 0.05	6.42	< 0.05
Norway	724	5389	0.12	0.02	37212	367	4823	0.07	0.17	36785	568	4804	33	0.07	0.17	36784	427.08	< 0.05	0.54	> 0.05
Oman	2860	6768	0.30	0.01	66282	1575	6438	0.20	0.17	65817	1655	6328	41	0.20	0.17	65816	465.35	< 0.05	0.69	> 0.05
Portugal	1581	3533	0.31	0.02	35941	644	3321	0.16	0.25	35609	616	3320	1	0.16	0.25	35609	332.56	< 0.05	0.05	> 0.05
Qatar	3876	4544	0.46	0.01	41716	2109	4344	0.33	0.24	41447	2011	4343	1	0.25	0.33	41447	268.64	< 0.05	0.09	> 0.05
Romania	3864	5338	0.42	0.01	47922	1641	4920	0.25	0.30	47434	1530	4909	23	0.25	0.30	47433	488.35	< 0.05	0.57	> 0.05
Russian federation	2743	3852	0.42	0.00	42831	1738	3769	0.32	0.15	42664	1832	3768	1	0.32	0.15	42664	166.46	< 0.05	0.14	> 0.05
Saudi Arabia	2759	4567	0.38	0.00	47658	1218	4478	0.21	0.20	47446	1394	4437	73	0.22	0.20	47442	212.78	< 0.05	3.87	> 0.05
Singapore	5872	1840	0.76	0.00	51230	1685	1840	0.48	0.55	50858	2071	1837	4	0.48	0.55	50854	371.80	< 0.05	4.44	> 0.05
South Africa	3848	2915	0.57	0.00	198626	1291	2913	0.31	0.33	198105	1509	2904	16	0.31	0.33	198099	521.10	< 0.05	5.06	> 0.05
Sweden	1382	4377	0.24	0.02	39570	411	3775	0.10	0.28	38887	766	3736	71	0.11	0.28	38884	683.27	< 0.05	2.26	> 0.05
Turkey	3886	7475	0.34	0.00	46082	1164	7020	0.14	0.27	45660	1782	7006	36	0.14	0.27	45659	422.15	< 0.05	1.49	> 0.05
United Arab Emirates	5915	3840	0.61	0.00	200878	3777	3798	0.50	0.23	200276	4054	3786	22	0.50	0.23	200274	602.17	< 0.05	2.31	> 0.05
United States	6172	3286	0.65	0.00	83959	2365	3220	0.42	0.40	83380	2455	3192	49	0.43	0.40	83376	578.58	< 0.05	4.36	> 0.05

The number of parameters in Model 1, Model 2 and Model 3 are 4, 8 and 10 respectively. Therefore, degrees of freedom in model comparisons between Model 1 and Model 2 are four and between Model 2 and Model 3 are two in in all countries

*ICC* interclass correlation, − *2LL* − 2 Loglikelihood
